# In vivo expression patterns of microRNAs of *Gallid herpesvirus* 2 (GaHV-2) during the virus life cycle and development of Marek’s disease lymphomas

**DOI:** 10.1007/s11262-015-1167-z

**Published:** 2015-02-11

**Authors:** Pu Zhao, Xiu-Jie Li, Man Teng, Lu Dang, Zu-Hua Yu, Jia-Qi Chi, Jing-Wei Su, Gai-Ping Zhang, Jun Luo

**Affiliations:** 1 College of Veterinary Medicine, Northwest A&F University, Yangling, 712100 People’s Republic of China; 2 Key Laboratory of Animal Immunology of the Ministry of Agriculture, Henan Provincial Key Laboratory of Animal Immunology, Henan Academy of Agricultural Sciences, No. 116 Huayuan Road, Zhengzhou, 450002 People’s Republic of China; 3 College of Animal Science and Veterinary Medicine, Henan Agricultural University, No. 63 Nongye Road, Zhengzhou, 450002 People’s Republic of China; 4 Department of Animal Science and Technology, He’nan Institute of Science and Technology, Xinxiang, 453003 People’s Republic of China; 5 Jiangsu Co-innovation Center for Prevention and Control of Important Animal Infectious Diseases and Zoonoses, Yangzhou, 225009 People’s Republic of China; 6 Present Address: College of Animal Science and Technology, Henan University of Science and Technology, Luoyang, 471003 People’s Republic of China

**Keywords:** MicroRNA, GaHV-2, MDV-1, Expression profile, Virus life cycle, Oncogenesis

## Abstract

In the past decade, a large number of microRNAs (miRNAs) have been identified in the viral genome of *Gallid herpesvirus* 2 (GaHV-2), which is historically known as Marek’s disease virus type 1. The biological role of most GaHV-2 miRNAs remains unclear. In the present study, we have performed an overall gene expression profile of GaHV-2 miRNAs during the virus life cycle at each phase of the developing disease, a highly contagious, lymphoproliferative disorder, and neoplastic immunosuppressive disease of poultry known as the Marek’s disease. According to their distinct in vivo expression patterns, the GaHV-2 miRNAs can be divided into three groups: 12 miRNAs in group I, including miR-M4-5p, displayed a typical expression pattern potentially correlated to the latent, late cytolytic, and/or the proliferative phases in the cycle of GaHV-2 pathogenesis; group II consisting of another 12 miRNAs with expression correlated to the early cytolytic and/or latent phases in GaHV-2’s life cycle; while the other two miRNAs in group III showed no identical expression features. Our findings may provide meaningful clues in the search for further potential functions of viral miRNAs in GaHV-2 biology.

*Gallid herpesvirus* 2 (GaHV-2), commonly known as Marek’s disease virus type 1 (MDV-1), is an oncogenic avian herpesvirus and classified as a member of the subfamily *Alphaherpesvirinae* [1]. GaHV-2 establishes and maintains latent infections in their natural hosts and may finally leads to a rapid-onset aggressive T cell lymphoma, an important neoplastic immunosuppressive disease of poultry named as Marek’s disease (MD) [2]. GaHV-2 infection provides an ideal model for investigating the biology, genetics, and immunology of virally induced tumors [3]. In the last decade, over 400 viral microRNAs (miRNAs) have been identified in diverse virus families [4], in particular those of the herpes viruses [5, 6], some of which have been demonstrated to potentially contribute to the control of lysis and latency, immune evasion, cell survival, proliferation, and oncogenic capabilities. Recently, a large numbers of miRNAs, which show a highly conserved genomic location, have been identified in the long or short repeat regions (R_L_/R_S_) of the viral genomes of GaHV-2, *Gallid herpesvirus* 3 (GaHV-3), and *Meleagrid* *herpesvirus* 1 (MeHV-1) [7–11].

In GaHV-2 genomes, a total of 26 miRNAs are encoded in 14 precursors and concentrated in three gene clusters, namely the Meq-cluster, the LAT-cluster, and the Mid-cluster [7, 8, 12], with their transcription driven by two distinct promoters [13, 14]. It is known that in miRNA biogenesis [15, 16], the primary miRNAs (pri-miRNAs) are mostly transcribed in a typical Pol II-dependent manner, to be processed into precursor miRNAs (pre-miRNAs). The precursors are transported from the nucleus to the cytoplasm, where they are cleaved by Dicer to produce the RNA duplexes, of which the functional miRNA is stably incorporated into an RNA-induced silencing complex (RISC) to regulate gene expression post-transcriptionally. Primary studies on GaHV-2 miRNAs have shown that most are expressed at higher levels in tumors and virally transformed T-lymphoma cell lines than in virus-infected chicken embryo fibroblast (CEF) [7, 8]. The miRNA of oncogenic GaHV-2 strains of differing virulence has highly conserved sequences, and the Meq-clustered miRNAs are more highly expressed in lymphomas induced by a very virulent plus (vv+) GaHV-2 strain than those produced by a very virulent (vv) GaHV-2 strain, whereas the LAT-clustered miRNAs have equal expression [17] suggesting that the Meq-clustered miRNAs may have a more significant role in GaHV-2 oncogenesis.

Up to now, only a few of the GaHV-2 miRNAs, such as miR-M4-5p [18–21], miR-M3-5p [22], and miR-M7-5p [23], have been shown to play critical roles in GaHV-2 replication, latency, pathogenesis, and/or oncogenesis. For most of the other viral miRNAs, their potential regulatory role in GaHV-2 biology remains speculative. In previous experiments [24], we have investigated the expression profiles of most viral miRNAs in very virulent GaHV-2 strain GX0101-challenged chickens during the late phases of disease and found that only a subset of GaHV-2 miRNAs was differentially expressed in diseased birds with obvious tissue-specific expression patterns. The course of MD is described in the standard ‘Cornell Model’ [25, 26]. This includes four phases: (1) the early cytolytic phase (2–7 dpi), (2) the latent phase (7–10 dpi onwards), (3) the late cytolytic phase (18 dpi onwards), and (4) the proliferative phase (28 dpi onwards). In the present study, we describe the expression profiles of all of the 26 GaHV-2 miRNAs during each stage in the cycle of MD pathogenesis, providing more information to help in unraveling their biological functions.

The animal experiments were conducted following the protocols of the Ethical and Animal Welfare Committee of Key Laboratory of Animal Immunology of the Ministry of Agriculture of China, simultaneously with those as we have previously reported [21]. Considering that our previous work [24] has shown that among eight different organs most GaHV-2 miRNAs are expressed at the highest levels in spleens, three birds challenged by GX0101 were randomly selected at 3, 7, 14, 21, 30, 45, and 60 days post-infection (dpi) for collecting the spleens and total RNAs were extracted using TRIzol reagent (Invitrogen) for subsequent experiments. Firstly, to evaluate the in vivo virus proliferation, gene expression levels of several representative GaHV-2 viral protein-coding genes in GX0101-infected chickens, including the unique oncogene *meq* (MDV *E* *co*RI-Q), the 38 KD phosphorylated protein gene (*pp38*), the structural glycoprotein B gene (*gB*), and the immediate-early (IE) infected cell protein 4 gene (*ICP4*), have been measured by quantitative real-time PCR (qRT-PCR). For cDNA synthesis, aliquots of 100 ng RNAs were first treated with RNase-free DNase I (TaKaRa) and then polyadenylated and reverse transcribed at 37 °C for 1 h in a 20 μl reaction mixture, using the NCode™ VILO™ miRNA cDNA Synthesis Kit (Invitrogen) according to the manufacturer’s instructions. The relative quantification of GaHV-2 viral protein-coding genes was performed. Briefly, a total 20 µl qPCR reaction was prepared, containing 10 μl of SuperMix Universal, 5 ng of cDNA, 50 nM of ROX Reference Dye (Invitrogen), and 200 nM of each primer specific for GaHV-2 genes (Table 1, primer pairs #1–#4). The 7500 Fast Real-Time PCR Systems (Applied Biosystems, Life Technologies, USA) was used for qPCR amplification with the reaction conditions set as follows: 95 °C for 3 min, 40 cycles of 95 °C for 3 s, and 60 °C for 30 s, followed by the thermal denaturing step to generate the dissociation curves to verify amplification specificity. Relative quantification of the mRNA gene expression was standardized to the level of endogenous chicken glyceraldehyde phosphate dehydrogenase (*GAPDH*) transcript determined using the same qPCR conditions with primer pair #5 (Table 1) and finally calculated with the $2^{{ - \Delta \Delta C_{t} }}$ method. Secondly, the absolute quantitation of GaHV-2 miRNAs was performed by qPCR as previously described [27]. The primers #6–#31, as listed in Table 1, specific for each GaHV-2 miRNA, were used as the forward primers for the qPCR analysis of miRNA expression using the same reactions and conditions as for the amplifications of viral protein-coding genes. Each of 200 nM of Universal qPCR Primer (Table 1, primer #32), provided by the NCode™ VILO™ miRNA cDNA Synthesis Kit (Invitrogen), was used as the reverse primer. Synthetic miRNA miR-M4-5p (GenePharma, Shanghai, China) was used as the standard to estimate the absolute miRNA copy number. Each reaction was repeated in triplicate and the values were used to calculate the means (M) ± standard deviations (SD), utilizing the software GraphPad Prism (version 5.0). The differences in expression levels of each viral protein-coding gene or miRNA between groups determined at distinct time point post-challenge were compared to 3 dpi and analyzed by one-way analysis of variance (One-way ANOVA, LSD) and were considered significant between groups at a probability level of *p* < 0.05.

**Table 1:** Primer pairs or primers used for the quantitative real-time PCR (qRT-PCR) analysis of the expression of viral miRNAs and GaHV-2 protein-coding genes in the present study

No.	Primer (pair)	Target	Type^a^	Sequence (5′–3′)^b^	Length (nt)
1	qPCRMeq-F	*meq*	5′	CGCAGGAAGCAGACGGACTA	20
	qPCRMeq-R	*meq*	3′	CCATAGGGCAAACTGGCTCAT	21
2	qMDV100-F	*ICP4*	5′	GCACTGCATTCCGAGAGTCAT	21
	qMDV100-R	*ICP4*	3′	TTGGGAATTTGGAGGGCG	18
3	qPCRgB-F	*gB*	5′	TCTAGGGCATGGCACACGAC	20
	qPCRgB-R	*gB*	3′	GAATACGGAAACACAGAGCGG	21
4	qPCRpp38-F	*pp38*	5′	CCGAAAGACAAAACCCAAAT	20
	qPCRpp38-R	*pp38*	3′	ATGTAACCAGCATATAAGAACGC	23
5	qPCRGAPDH-F	*GAPDH*	5′	AAGTCCCTGAAAATTGTCAGCAAT	24
	qPCRGAPDH-R	*GAPDH*	3′	ATGGCATGGACAGTGGTCATAAG	23
6	miR-M1-3p-F	miR	5′	GCGCATGAAAGAGCGAAAA	19
7	miR-M1-5p-F	miR	5′	TGTTCACTGTGCGGCAAAAA	20
8	miR-M2-3p-F	miR	5′	CTGCCGCAGAATAGCTTAAAAA	22
9	miR-M2-5p-F	miR	5′	GTTGTATTCTGCCCGGTAGTCC	22
10	miR-M3-3p-F	miR	5′	GGGGGGTTCACATTTTTAAGTAAA	24
11	miR-M3-5p-F	miR	5′	TGAAACCTCTCCCGCAAAAA	20
12	miR-M4-3p-F	miR	5′	GGTTCTGACAGCATGACCAAAAA	23
13	miR-M5-3p-F	miR	5′	TGTGTATCGTGGTCGTCTACTGTAAA	26
14	miR-M4-5p-F	miR	5′	TTAATGCTGTATCGGAACCCTTC	23
15	miR-M5-5p-F	miR	5′	CGTATGCGATCACATTGACAAAA	23
16	miR-M6-3p-F	miR	5′	GATCCCTGCGAAATGACAGTAAA	23
17	miR-M6-5p-F	miR	5′	TCTGTTGTTCCGTAGTGTTCTCAAA	25
18	miR-M7-3p-F	miR	5′	TCGAGATCTCTACGAGATTACAGAAAA	27
19	miR-M7-5p-F	miR	5′	CGGGGAGATCCCGATAAAAA	20
20	miR-M8-3p-F	miR	5′	GTGACCTCTACGGAACAATAGTAAAAA	27
21	miR-M8-5p-F	miR	5′	TATTGTTCTGTGGTTGGTTTCGA	23
22	miR-M9-3p-F	miR	5′	CGAGGGCAGGAAAAAGAAAAA	21
23	miR-M9-5p-F	miR	5′	CTTCCCCCCGGAGTTAAAAA	20
24	miR-M10-3p-F	miR	5′	TCGAAATCTCTACGAGATAACAAAAA	26
25	miR-M10-5p-F	miR	5′	TTGTCTCGTAGAGGTCCAGAAAAA	24
26	miR-M11-3p-F	miR	5′	AGTTACATGGTCAGGGGATTAAAAA	25
27	miR-M11-5p-F	miR	5′	TTTTCCTTACCGTGTAGCTTAGAAA	25
28	miR-M12-3p-F	miR	5′	TGCATAATACGGAGGGTTCTAAAA	24
29	miR-M12-5p-F	miR	5′	GCCCTCCGTATAATGTAAATGTAAAA	26
30	miR-M13-3p-F	miR	5′	ATGGAAACGTCCTGGGAAAAA	21
31	miR-M31-3p-F	miR	5′	CTACAGTCGTGAGCAGATCAAAAA	24
32	Universal qPCR Primer	miR	3′	UA	

*miR* miRNA, *GAPDH* glyceraldehyde phosphate dehydrogenase, *meq* MDV *E* *co*RI-Q, *ICP4* immediate-early gene *ICP4* (infected cell protein 4), *gB* virion membrane glycoprotein B, *pp38* 38 KD phosphorylated protein

^a^5′, forward primer; 3′, reverse primer

^b^UA, the Universal qPCR Primer is provided by Invitrogen with unavailable sequence

Using the pattern of disease progress described in the ‘Cornell Model’ [25, 26], the GaHV-2 protein-coding genes, including *gB*, *ICP4*, *pp38,* and *meq*, gave expression patterns as expected in the spleens of GX0101-challenged birds during the early stages of GaHV-2 infection (Fig. 1). The relative expression levels of all four viral genes increased first at 7 dpi (the early cytolytic phase), decreased at 14 dpi (the latent phase), and then peaked at 21 dpi (the late cytolytic phase). At the late time period of 30–60 dpi (the transformation and proliferative phase), the expressions of the genes of *gB*, *ICP4*, and *pp38* were gradually reduced (Fig. 1a–c) but *meq* remained expressed at a higher level (Fig. 1d), reflecting its role as a viral oncogene [26].

**Fig. 1:**
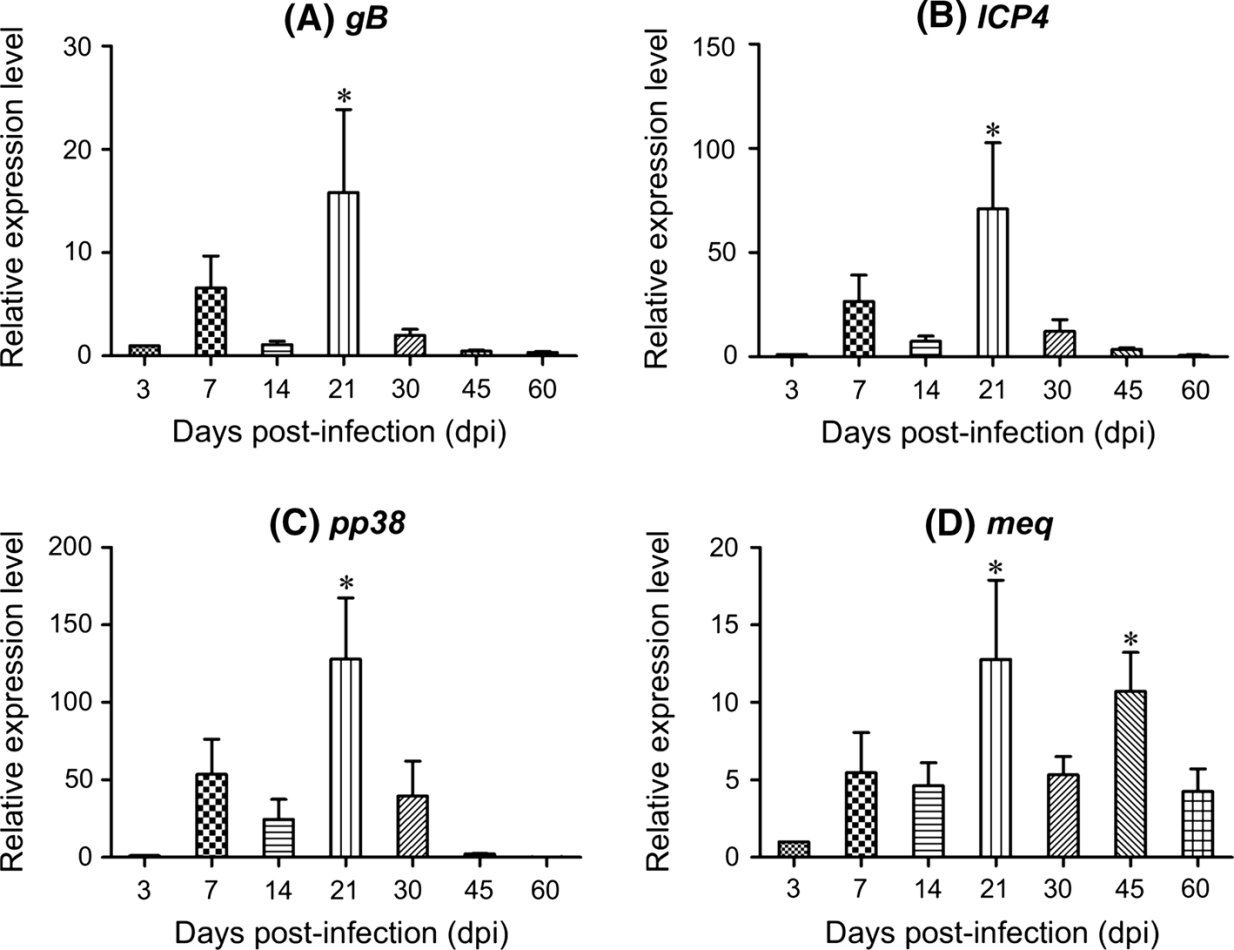
Relative expression levels of the GaHV-2 protein-coding genes analyzed by qRT-PCR. **a** *gB*, the gene of glycoprotein B; **b** *ICP4*, the gene of infected cell protein 4; **c** *pp38*, the gene of 38 KD phosphorylated protein; **d** *Meq*, the oncogene of MDV *E* *co*RI-Q. The endogenous chicken *GAPDH* gene was used as a standard and the relative quantification of mRNA expression was calculated with the $2^{{ - \Delta \Delta C_{t} }}$ method. *Columns* represent the mean of relative expression levels in the spleens of three randomly selected birds, determined in triplicate. *Error bars* indicate 1× SD. The *star* indicates significant difference (*p* < 0.05) compared to that determined at 3 dpi

However, distinct from the viral protein-coding genes, the GaHV-2 miRNAs could be divided into three groups with different temporal expression profiles during the progress of the disease. The expression levels of 12 viral miRNAs, including the miR-M4-5p, miR-M2-5p, miR-M2-3p, miR-M5-3p, miR-M3-3p, and miR-M12-3p in the Meq-cluster (Fig. 2a); the miR-M8-5p, miR-M8-3p, miR-M6-5p, miR-M6-3p, and miR-M7-3p in the LAT-cluster (Fig. 2b); and the miR-M11-5p in the Mid-cluster (Fig. 2c) increased and reached a first peak at 14–21 dpi, decreased by 30 dpi, and were then highly expressed again at 45–60 dpi. These miRNAs constitute the first group and they displayed an expression pattern that may be closely correlated to the latent, late cytolytic, and/or the proliferative phases in MD pathogenesis. One of the highest expressed GaHV-2 miRNAs, miR-M4-5p, has been characterized as a functional miR-155 ortholog [18], and deletion of miR-M4 from the viral genomes of the virulent (v) GaHV-2 strain pRB-1B5 and the very virulent GaHV-2 strain GX0101 significantly decrease their oncogenicity [20, 21]. In addition to the previously reported conserved cellular genes targeted by cellular miR-155 [28], we have shown recently that miR-M4-5p down-regulates the expression of the latent transforming growth factor-β (TGF-β)-binding protein 1 (LTBP1). This leads to a significant decrease in activation and secretion of TGF-β1, with concomitant suppression of TGF-β signaling and a significant increase in the level of expression of c-MYC, a well-known oncogene which is critical for the virally induced tumorigenesis [29]. In addition to its involvement in GaHV-2 oncogenesis, miR-M4-5p has also been shown to specifically inhibit the production of UL28 [19], a viral protein homologous to a human herpesvirus 1 (HHV-1) protein involved in the cleavage/packaging of viral DNA. These facts help to explain the expression pattern of miR-M4-5p during GaHV-2’s life cycle and in particular its higher expression level during the late stages of the developing disease. The other 11 miRNAs of group I displayed expression patterns similar to that of miR-M4-5p. We have recently shown that in addition to miR-M4-5p, deletions of the other individual Meq-clustered miRNAs, i.e., miR-M2, miR-M3, and miR-M12, also variably decrease the pathogenicity and oncogenicity of the virus [30]. Thus, whether or not these miRNAs encoded in the Meq or other two clusters play a similar part in the virus life cycle remains to be further studied.

**Fig. 2:**
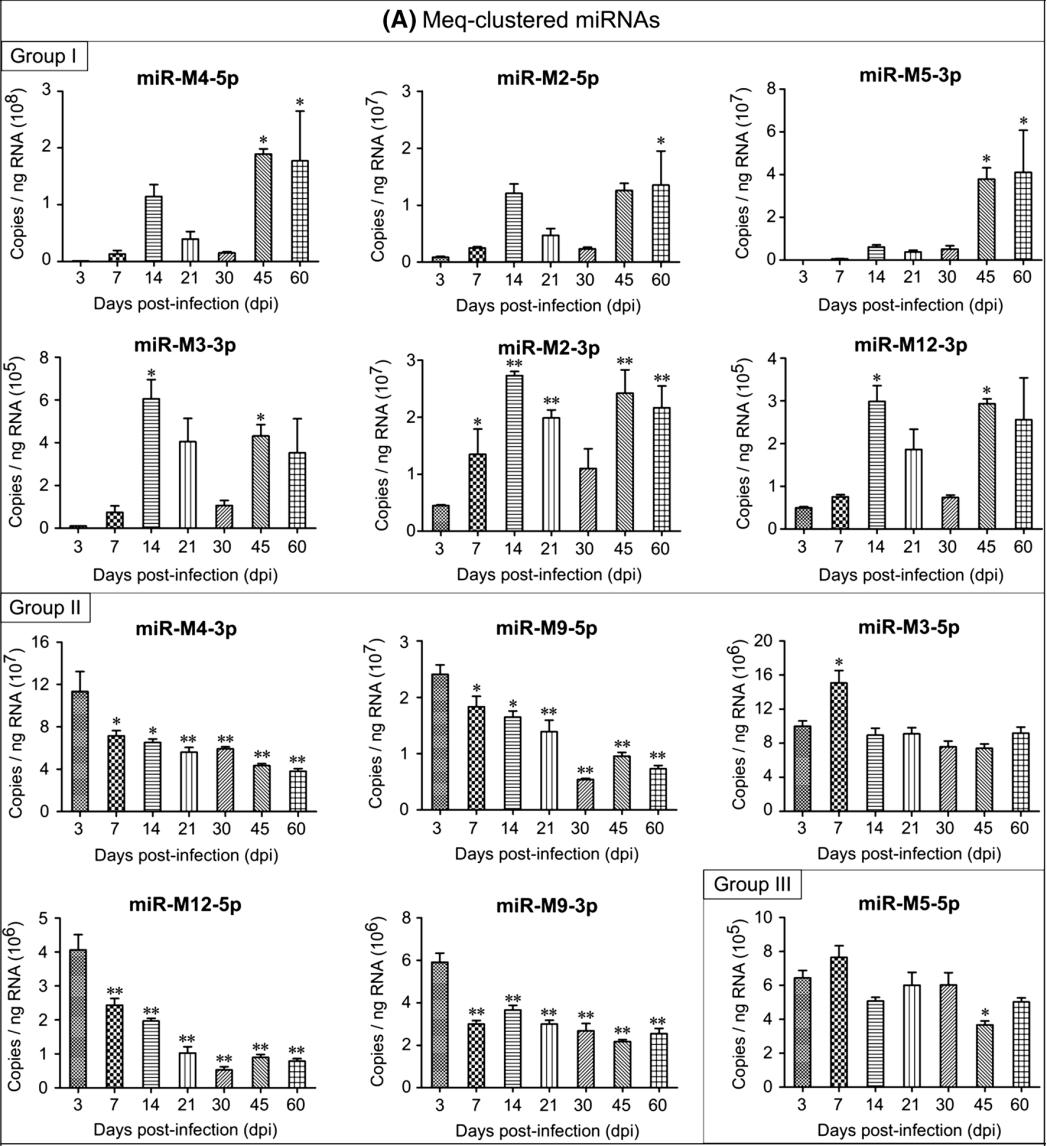

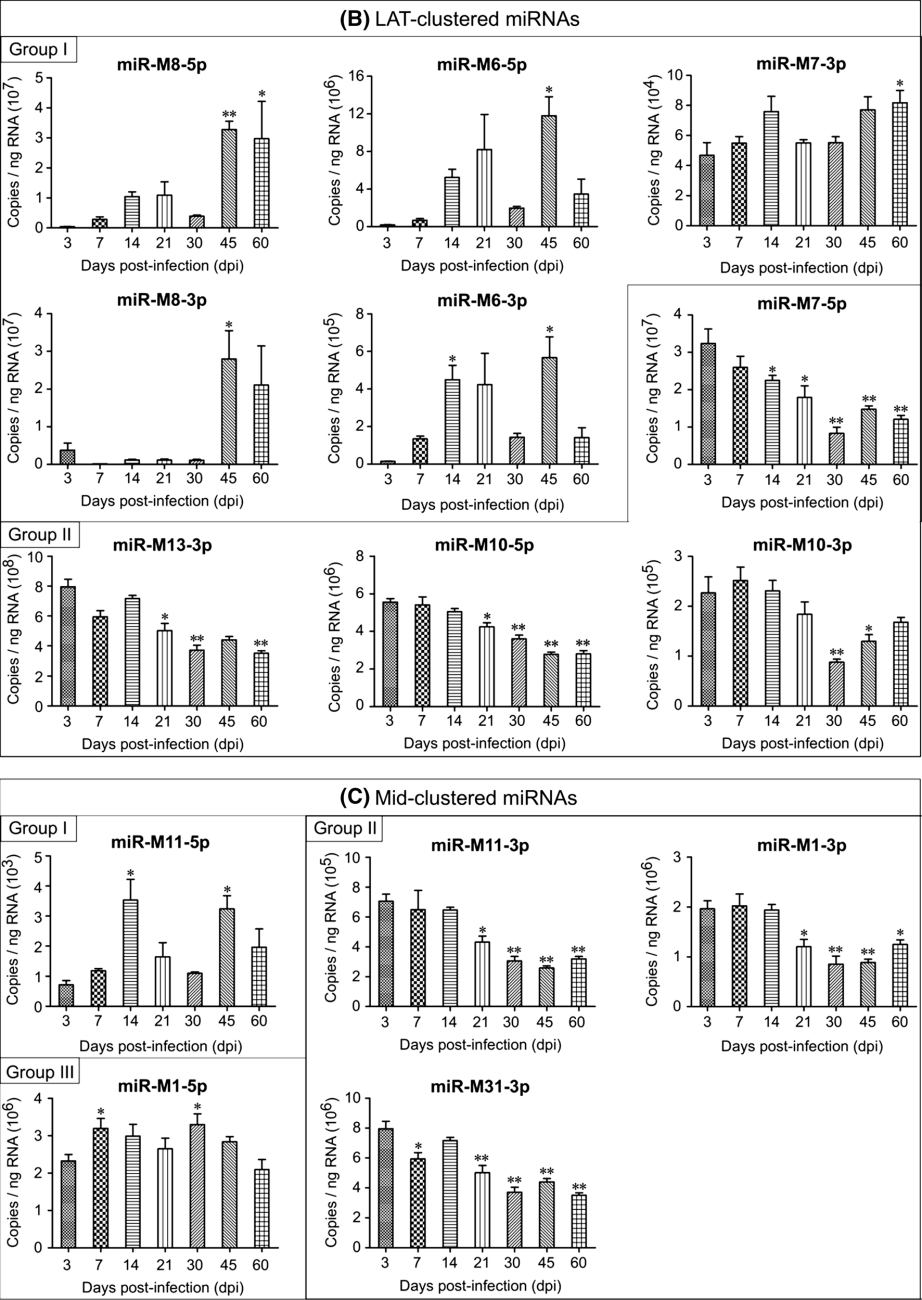
Expression profiles of GaHV-2 miRNAs during the different phases of the developing disease. **a** The Meq-clustered miRNAs; **b** the LAT-clustered miRNAs; **c** the Mid-clustered miRNAs. A quantitative real-time PCR was performed to determine miRNA expression and the absolute copy number was estimated using synthetic miR-M4-5p as the standard. *Columns* represent the mean miRNA expression levels in the spleens from three randomly selected birds, determined in triplicate. *Error bars* indicate 1× SD. The single and double stars indicate significant differences (*p* < 0.05 or *p* < 0.01) compared to that determined at 3 dpi

The other 12 GaHV-2 miRNAs showing a different expression pattern include miR-M4-3p, miR-M12-5p, miR-M9-5p, miR-M9-3p, and miR-M3-5p in the Meq-cluster (Fig. 2a); the miR-M7-5p, miR-M10-5p, miR-M10-3p, and miR-M13-3p in the LAT-cluster (Fig. 2b); and the miR-M1-3p, miR-M11-3p, and miR-M31-3p in the Mid-cluster (Fig. 2c) and constitute the second group. Most of these miRNAs were expressed at higher levels during the first 1–2 weeks post-challenge with subsequent low levels of expression, although some display a slight increase at 45 or 60 dpi. Based on these results, it seems that the miRNAs included in group II are more closely correlated to the early cytolytic and/or the latent phases in GaHV-2’s life cycle. Such a suggestion may be further supported in part by some recent reports. The miR-M4-3p has been shown to specifically target and repress UL32 production [19], another viral protein homologous to a HHV-1 protein involved in the cleavage/packaging of herpes virus DNA. A recent study has also demonstrated that miR-M7-5p, located in the first intron of the latency-associated transcripts (LATs), targets the immediate-early genes *ICP4* and *ICP27* [23], providing a possibly contribution to the establishment and/or maintenance of latency. Furthermore, the miR-M3-5p, which was highly expressed particularly at 7 dpi, has been shown to target and down-regulate the expression of Smad2 [22], a critical component in TGF-β signal pathway, potentially creating a cellular environment beneficial to viral latency. Thus, the possibility of similar roles for the other GaHV-2 miRNAs of group II may be uncovered in future studies.

However, the other two miRNAs, miR-M5-5p in the Meq-cluster (Fig. 2a) and miR-M1-5p in the Mid-cluster (Fig. 2c), display no identical expression features, constitute a third group (group III). Previous studies have shown that the transcription of GaHV-2 miRNAs located in both of the Meq- and Mid-clusters is driven by a single promoter ‘prmiRM9M4’ [13], whereas that of all the LAT-clustered miRNAs are driven by a p53-dependent promoter [14]. It is interesting that during the developing of disease, some of the mature miRNAs processed from a same pre-miRNAs (such as miR-M2, miR-M9, miR-M6, miR-M8, and miR-M10) display similar expression profiles, while the other miRNAs represent expression patterns different from each other. Such a phenomenon has also been observed among miRNAs that had been identified in diverse species, such as in animals, plants, and even in viruses, but little of the underlined precious mechanism is known to date. In addition, we also observed that the expression patterns of miR-M9-5p and miR-M7-5p presented here were not in accordance with our previous report [24]. Whether it was caused by the differential methodology, primer or other factors need to be further studied.

In recent years, identifications of miRNA expression signatures in many cancers have increased our further understanding of the connection between miRNA and cancer [31]. Our present research on the in vivo expression profiles of GaHV-2 miRNAs may provide meaningful clues for the prediction, selection, and characterization of their mRNA targets, and further evaluating their biological functions in the virus life cycle and development of MD tumors and other cancers. Based on the successful establishment of strategies for mRNA target prediction and subsequent experimental identification along with the application of bacterial artificial chromosomes (BAC) in research on MD pathogenesis, focus on the miRNAs highlighted in this study should reveal more about their roles.
